# Carcinoma Cuniculatum Masquerading as Eumycetoma: An Unacquainted Entity Posing a Diagnostic Dilemma

**DOI:** 10.5146/tjpath.2024.13373

**Published:** 2025-05-31

**Authors:** Pooja Sharma, Pragya Jain, Ankur Garg, Sonal Sharma

**Affiliations:** Department of Pathology, University College of Medical Sciences & Guru Teg Bahadur Hospital, Delhi, India; Department of Surgery, University College of Medical Sciences & Guru Teg Bahadur Hospital, Delhi, India

**Keywords:** Carcinoma cuniculatum, Verrucous carcinoma, Squamous cell carcinoma, Eumycetoma

## Abstract

Carcinoma cuniculatum (CC) is a rare and distinct clinicopathological variant of well-differentiated squamous cell carcinoma. It is a rare and slow-growing tumor with a peculiar infiltrative growth pattern resembling rabbit burrows (cuniculi). It usually occurs over the plantar aspect of the foot but can also occur at other sites like the oral cavity and genitals. The pathogenesis is unknown, with various hypotheses of trauma as proposed by different authors. It is essential to be aware of this entity as it commonly mimics benign and other low-grade squamous cell carcinoma (SCC). Diagnosis of CC can be challenging and requires repeated histological evaluation and clinical correlation. Herein, we present a case report of CC of the plantar and dorsal aspect of the foot in a 60-year-old male with a history of multiple chronic non-healing ulcers, which was clinically suspected as eumycetoma and remained inconclusive on numerous biopsies.

## INTRODUCTION

Carcinoma cuniculatum (CC) is a rare, distinct clinicopathological variant of well-differentiated squamous cell carcinoma of the skin. Arid et al. first described it in 1954 over the plantar aspect of the foot (1). Apart from the plantar part of the foot, other potential sites include the oral cavity, genitalia, upper-aerodigestive tract, larynx, etc (2–4). The tumor has a peculiar infiltrative growth pattern resembling rabbit burrows, hence, the name cuniculatum (Latin word for rabbit warren). It is a slow-growing, locally destructive tumor showing infiltration into the deep dermis and bone. It rarely metastasizes and does not involve locoregional lymph nodes (5). To the best of our knowledge, only 100 cases are reported in the literature worldwide, with only a few cases from India (3,6). Due to its rarity and paucity of literature, clinicians and pathologists are unaware of this entity, leading to delays in diagnosis. Furthermore, it often masquerades clinically as a benign lesion such as keratocyst, osteomyelitis, epidermal cyst, or even eumycetoma (7–10). Herein, we reported a case with a long history of multiple non-healing ulcers on the foot’s plantar and dorsal aspects and that was clinico-radiologically suspected to be eumycetoma.

## CASE PRESENTATION

**Figure 1:**
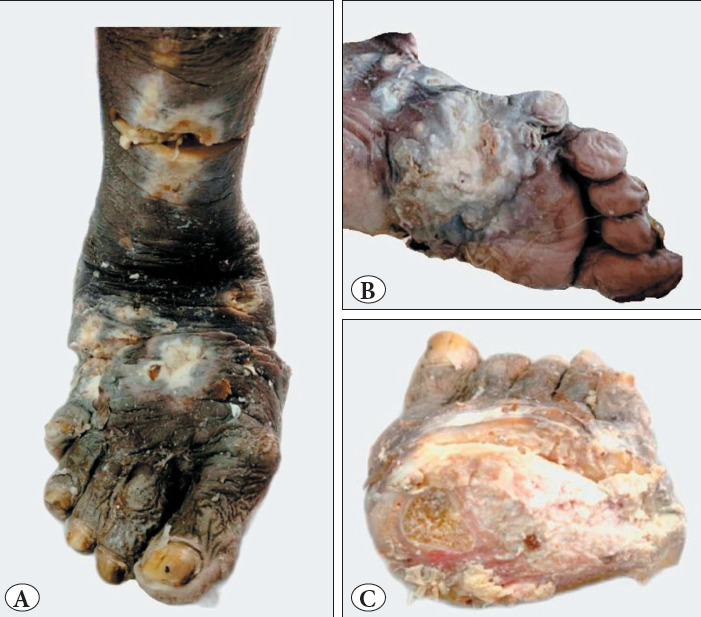
**A)** Amputated lower limb showing multiple ulcers at the dorsal aspect of the foot and the anterior aspect of the leg. **B)** An ulceroproliferative growth of yellowish-white base seen involving the dorsum of the foot. **C)** Coronal section showing the destruction of all the metatarsal bones with the erosion of the surface of the 1st metatarsal bone.

A 60-year-old male presented to surgery OPD for multiple non-healing ulcers over the right foot and leg for four years with malodorous pus discharge for nine months. The patient underwent multiple debridement for ulcers; however, the ulcers remained non-healing. Previous biopsies showed dense, acute on chronic inflammation with keratin debris for which an infective pathology was suggested. Because of the long-standing history of deep non-healing ulcers and foul-smelling whitish-yellow pus discharge, a clinico-radiological diagnosis of eumycetoma was considered, and a below-knee amputation of the right leg was done. Our histopathological department received an amputated limb specimen (Figure 1) comprising the right lower leg measuring 32 cm in length and the foot measuring 22 cm in length. A deep ulcer was present on the plantar aspect of the lateral side of the foot, measuring 5 x 4 cm at 10 cm from the lateral malleolus. In addition, multiple irregular ulcers of varying sizes were present on the dorsum of the foot, showing a yellowish-white base. On bone sectioning, all tarsal and metatarsal bones except the 1st metatarsal were destroyed and replaced by whitish growth.

**Figure 2:**
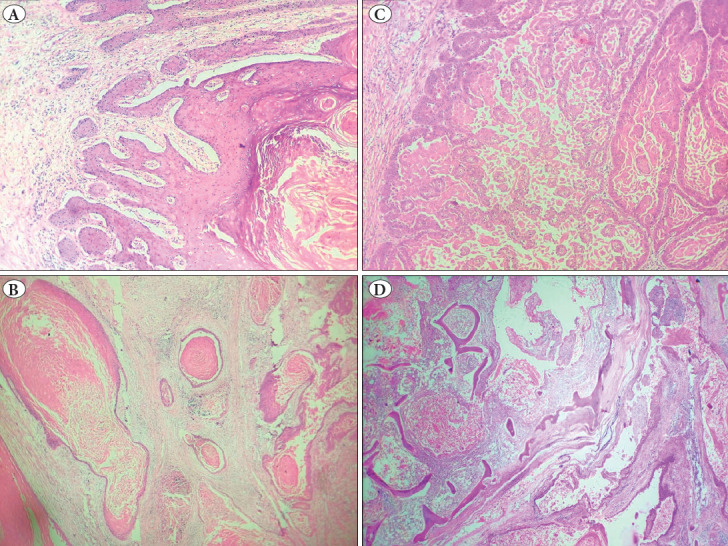
Histology of carcinoma cuniculatum. **A)** Sinus is lined by squamous epithelium showing pseudoepitheliomatous hyperplasia with low-grade cytologic atypia. **B)** Sinuses are filled with keratin flakes and some acantholytic cells. **C)** Multiple confluent sinuses in a burrowing pattern. **D)** Multiple deep sinuses causing bone destruction.

**Figure 3:**
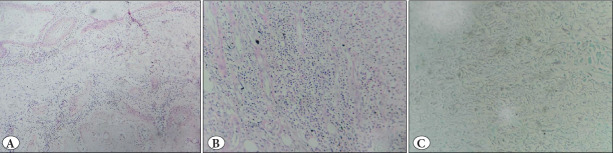
Special stains for bacterial & fungal elements. **A)** Gram Stain. **B)** PAS Stain. **C)** Grocott methenamine silver (GMS) stain

Hematoxylin and Eosin-stained sections (H&E) from the foot showed multiple, long, deep infiltrating sinuses in a burrowing pattern (Figure 2). These sinuses displayed pseudoepitheliomatous hyperplasia with low cytological atypia and were filled with keratinous debris and many acantholytic cells. They were seen to extend up to the underlying muscular compartment and cartilage with the destruction of bone by the keratinous debris. Ulcers on the anterior aspect of the leg showed acute to chronic inflammation with no evidence of malignancy in those sections. The stains for bacterial and fungal organisms were negative (Figure 3). The skin, soft tissue, and bony margins were free of the tumor with no evidence of vascular/neural/lymph node invasion.

Given the above histopathological findings, a final diagnosis of well-differentiated squamous cell carcinoma (cuniculatum variant) was made.

The patient was re-evaluated for regional/distant metastasis after a diagnosis of carcinoma cuniculatum but no regional/distant metastasis was found on computer tomography and the patient did not receive any adjuvant therapy. The post-operative period was uneventful, and the patient was discharged subsequently. The patient was kept on follow-up for a year during which no signs of infection or recurrence of the disease were found.

## DISCUSSION

CC is a potentially rare variant of cutaneous squamous cell carcinoma, first reported on the plantar aspect of the foot as an ulceroproliferative growth (1). It presents more commonly in males, usually in the sixth decade of life (11). Various names and eponyms describe it at different sites like epithelioma cuniculatum, inverted verrucous carcinoma, Ackermann tumor, Buschke-Lowenstein tumor, Cutis papillomatosis carcinoides of Gottron, etc. Due to the difference in histology and prognosis, the WHO in 2005 recognized it as a separate variant of squamous cell carcinoma (SCC) in malignant tumors of the oral cavity and oropharynx, but it is still included and discussed under verrucous carcinoma at other sites (12). Farag et al. reviewed cases of oral CC reported till 2018. They cited 17 studies with a total of 43 cases of CC in the oral cavity. Almost all had a similar presentation with penetration deep into the underlying soft tissue and bone in the form of deep burrows and crypts filled with keratinous debris and purulent material. Preoperative diagnosis of osteomyelitis, abscess, keratocyst, pseudoepitheliomatous hyperplasia, or verrucous carcinoma (VC) led to confusion (4).

Although the pathogenesis remains unknown, various etiologies are being suggested like chronic and repeated plantar traumatism (as in our case), slowly repairing bone fracture or osteomyelitis, local infiltration of corticosteroids, chronic decubitus ulcer, chronic inflammatory diseases (e.g.-plantar keratoderma, plantar intertrigo), and viral infections like HPV (11,13,14).

A superficial biopsy remains inconclusive in most cases, as a pathologist cannot comment upon invasion in such cases. Various case studies have been reported in the past literature, where it has masqueraded as a benign lesion on presurgical histopathology Table 1.

In our case, a deeper biopsy was attempted, which revealed extensive pseudoepitheliomatous hyperplasia with numerous acantholytic cells and keratin debris. Still, no definitive comment on malignancy was made due to the lack of invasion.

It is imperative to distinguish this entity from other potential benign and malignant mimics such as plantar wart (verruca vulgaris), keratoacanthoma, pseudoepitheliomatous hyperplasia due to chronic osteomyelitis, or draining sinuses of eumycetoma infection and verrucous carcinoma. Therefore, carefully considering subtle differentiating points and an algorithmic approach may lead to an accurate and timely diagnosis.

Verruca vulgaris (VV) is smaller, does not ulcerate commonly, and shows marked hyperkeratosis, acanthosis, and papillomatosis with koilocytes in the superficial dermis. Deep sinus tracts are lacking in this entity (15). Keratoacanthomas share similar architectural features as VV but have a central crater lacking in our case (15). Slow-healing osteomyelitis due to infectious etiology such as tuberculosis may present as multiple draining sinuses on the skin surface. On histopathology, they also show pseudoepitheliomatous hyperplasia with underlying bone destruction by inflammatory exudate. However, the pseudoepitheliomatous hyperplasia is not so extensive in such cases, and granuloma with non-confluent sinuses generally points toward chronic tubercular infection. Such extensive bone destruction is also not seen. Moreover, pseudoepitheliomatous hyperplasia usually shows uneven, sharply pointed, and jagged-down growths, while our case had rounded rete ridges (16). Eumycetoma infection also shows discharging sinuses reaching deep into subcutaneous tissue with typical filamentous bacteria on gram staining. Stain for bacterial and fungal organisms was negative in our case, ruling out the possibility of infection.

It is essential to differentiate it from other histological subtypes of SCC, such as VC and different invasive patterns with a low degree of worst pattern of invasion (WPOI). VC shows both exophytic and endophytic architecture with deep tongues of intradermal growth in a club-shaped manner without a burrowing growth pattern (15). Invasive SCC with low WPOI generally shows invading foci with significant dysmaturation and locoregional lymph node involvement. CC predominantly presents as an endophytic lesion and spreads within the subepithelium by deep burrowing sinuses. Despite the lack of definitive stromal invasion, this justifies its placement in well-differentiated carcinoma.

Due to its rarity, adequate literature on the prognosis and treatment is lacking. The mainstay of treatment is complete surgical excision with acceptable safety margins. More extensive surgery is required in case of extensive bone erosion and destruction. Other conservative therapeutic approaches such as electrodesiccation, cryotherapy, and laser ablation are not always curative and may lead to tumor recurrence (10,11,17–19). Nevertheless, they generally have an excellent prognosis with good disease-free duration. Recurrence is noted in very few cases, which may be due to inadequate tumor removal due to preoperative diagnosis as a benign lesion. The prognosis is good and is intermediate between VC and invasive SCC, i.e., better than SCC but poorer than VC (4).

**Table 1:** Literature review for clinical mimickers of carcinoma cuniculatum on the foot

**S. No.**	**Case report**	**Age**	**Site**	**Clinical diagnosis**	**Treatment**
1	Mendez-Ojeda et al. (10)	76/F	The anterior sole of the right foot	Plantar wart	Amputation with a wide margin
2	Vlahovic et al. (11)	70/M	Left fifth metatarsal head	Plantar wart/diabetic ulcer	Full-thickness excision
3	Shenoy et al. (18)	56/M	The ball of the right foot	Ulceroproliferative growth	Surgical excision
4	Mohamed et al. (19)	55/M	Non-healing nodule on the sole	Plantar callus	Trans metatarsal amputation
5	Mckay et al. (20)	77/M	Third and fourth web spaces of his left foot	Intractable toe web intertrigo	Amputation of the second, third, and fourth toes

## CONCLUSION

To summarize, the diagnosis of CC should be suspected in a long-standing non-healing ulcer with verrucous/ulceroproliferative growth and malodorous pus discharge with histological features of deep infiltrating sinuses filled with keratinous material and lined by squamous epithelium showing low-grade cytological atypia. A deeper biopsy, when clinical judgment warrants it, and a clinico-radiological correlation are mandatory to arrive at an accurate diagnosis.

## Conflict of Interest

The authors declare that they have no conflict of interest for this article.

## References

[ref-1] Aird I., Johnson H. D., Lennox B., Stansfeld A. G. (1954). Epithelioma cuniculatum: a variety of squamous carcinoma peculiar to the foot. Br J Surg.

[ref-2] Elangovan Elampavai, Banerjee Abhishek, Abhinandan null, Roy Bireswar (2021). Oral carcinoma cuniculatum. J Oral Maxillofac Pathol.

[ref-3] Thavaraj Selvam, Cobb Alistair, Kalavrezos Nicholas, Beale Timothy, Walker Donald Murray, Jay Amrita (2012). Carcinoma cuniculatum arising in the tongue. Head Neck Pathol.

[ref-4] Farag Amina Fouad, Abou-Alnour Dalia Ali, Abu-Taleb Noha Saleh (2018). Oral carcinoma cuniculatum, an unacquainted variant of oral squamous cell carcinoma: A systematic review. Imaging Sci Dent.

[ref-5] Fugate D. S., Romash M. M. (1989). Carcinoma cuniculatum (verrucous carcinoma) of the foot. Foot Ankle.

[ref-6] Kunc Michał, Biernat Wojciech (2019). Carcinoma Cuniculatum of the Lower Leg: A Case Report and Proposed Diagnostic Criteria. Am J Dermatopathol.

[ref-7] Janardhanan Mahija, Rakesh S., Savithri Vindhya, Aravind Thara, Mohan Mridula (2021). Carcinoma Cuniculatum of Mandible Masquerading as Odontogenic Keratocyst: Challenges in the Histopathological Diagnosis. Head Neck Pathol.

[ref-8] Arisi Mariachiara, Zane Cristina, Edu Irina, Battocchio Simonetta, Petrilli Giulia, Calzavara-Pinton Pier Giacomo (2016). Carcinoma Cuniculatum of the Foot Invading the Bone Mimicking a Pseudo-Epitheliomatous Reaction to an Acute Osteomyelitis. Dermatol Ther (Heidelb).

[ref-9] Arefi Mahbod, Philipone Elizabeth, Caprioli Russell, Haight John, Richardson Hugh, Sheng Chen null (2008). A case of verrucous carcinoma (epithelioma cuniculatum) of the heel mimicking infected epidermal cyst and gout. Foot Ankle Spec.

[ref-10] Méndez-Ojeda Mm, Corona Pérez-Cardona P, Herrera-Pérez M, Pais-Brito J (2021). Epitelioma cuniculatum de la planta del pie simulando una infección. Acta Ortopédica Mexicana.

[ref-11] Vlahovic Tracey C., Klimaz Tracy L., Piemontese Maria K., Zinszer Kathya M. (2009). Plantar verrucous carcinoma: an unusual case of bone invasion and osteomyelitis. Adv Skin Wound Care.

[ref-12] Datar Uma Vasant, Kale Alka, Mane Deepa (2017). Oral Carcinoma Cuniculatum: A New Entity in the Clinicopathological Spectrum of Oral Squamous Cell Carcinoma. J Clin Diagn Res.

[ref-13] Feng Chin-Jung, Li Wing-Yin, Liu Han-Nan, Ma Hsu, Wu Szu-Hsien (2016). Carcinoma cuniculatum of the nasal tip. Formosan Journal of Surgery.

[ref-14] Noel J. C., Peny M. O., Detremmerie O., Verhest A., Heenen M., Thiry L., De Dobbeleer G. (1993). Demonstration of human papillomavirus type 2 in a verrucous carcinoma of the foot. Dermatology.

[ref-15] Elder DE, Massi D, Scolyer RA, Willemze R (2018). WHO Classification of Skin Tumours.

[ref-16] Headington JT (1978). Verrucous carcinoma.

[ref-17] Thomas Eric J., Graves Nathan C., Meritt Stephen M. (2014). Carcinoma cuniculatum: an atypical presentation in the foot. J Foot Ankle Surg.

[ref-18] Shenoy Asha S., Waghmare Ramesh S., Kavishwar Vikas S., Amonkar Gayathri P. (2011). Carcinoma cuniculatum of foot. Foot (Edinb).

[ref-19] Mohamed Mariem, Belhadjali Hichem (2014). Carcinoma cuniculatum of the foot arising on plantar callus. Pan Afr Med J.

[ref-20] McKay Catherine, McBride Penelope, Muir James (2012). Plantar verrucous carcinoma masquerading as toe web intertrigo. Australas J Dermatol.

